# Multi-Camera-Based Human Activity Recognition for Human–Robot Collaboration in Construction

**DOI:** 10.3390/s23156997

**Published:** 2023-08-07

**Authors:** Youjin Jang, Inbae Jeong, Moein Younesi Heravi, Sajib Sarkar, Hyunkyu Shin, Yonghan Ahn

**Affiliations:** 1 Department of Civil, Construction and Environmental Engineering, North Dakota State University, Fargo, ND 58108, USA; moein.younesiheravi@ndsu.edu (M.Y.H.); sajib.sarkar@ndsu.edu (S.S.); 2 Department of Mechanical Engineering, North Dakota State University, Fargo, ND 58108, USA; inbae.jeong@ndsu.edu; 3 Sustainable Smart City Convergence Educational Research Center, Hanyang University ERICA, Ansan 15588, Republic of Korea; hyunkew@hanyang.ac.kr; 4 Department of Architectural Engineering, Hanyang University ERICA, Ansan 15588, Republic of Korea; yhahn@hanyang.ac.kr

**Keywords:** human pose estimation, human activity recognition, multiple cameras, particle filter, long short-term memory

## Abstract

As the use of construction robots continues to increase, ensuring safety and productivity while working alongside human workers becomes crucial. To prevent collisions, robots must recognize human behavior in close proximity. However, single, or RGB-depth cameras have limitations, such as detection failure, sensor malfunction, occlusions, unconstrained lighting, and motion blur. Therefore, this study proposes a multiple-camera approach for human activity recognition during human–robot collaborative activities in construction. The proposed approach employs a particle filter, to estimate the 3D human pose by fusing 2D joint locations extracted from multiple cameras and applies long short-term memory network (LSTM) to recognize ten activities associated with human and robot collaboration tasks in construction. The study compared the performance of human activity recognition models using one, two, three, and four cameras. Results showed that using multiple cameras enhances recognition performance, providing a more accurate and reliable means of identifying and differentiating between various activities. The results of this study are expected to contribute to the advancement of human activity recognition and utilization in human–robot collaboration in construction.

## 1. Introduction

To enhance productivity and address labor shortage and safety concerns, the construction industry is at the forefront of embracing a range of modern automation technologies. Among these, robots stand out as one of the most promising technologies capable of revolutionizing the construction landscape. These mechanical marvels possess the remarkable ability to carry out repetitive or hazardous construction activities with unparalleled speed, strength, and safety, thereby significantly increasing overall productivity and reducing fatalities [1,2,3]. However, the unique cognitive skills possessed by human workers remain indispensable for certain intricate tasks that demand adaptability and ingenuity. For instance, during drywall installation, a robot may encounter novel challenges that require fine-tuning the drywall panel or adjusting nailing angles, tasks that demand the quick thinking and problem-solving prowess of human workers. Recognizing the immense potential of collaboration between human workers and robots, the construction industry is now actively pursuing close-proximity human–robot teamwork as a key strategy to harness the complementary strengths of both. By promoting harmonious collaboration between these two forces, the construction industry aims to unlock new synergies and optimize construction tasks, ultimately achieving a seamless fusion of human expertise and robotic efficiency [4,5].

Nonetheless, ensuring the safety and productivity of human workers in collaborative environments presents a significant challenge. Collisions between human workers and robots can lead to work disruptions, robot malfunctions, and, more critically, severe injuries. Establishing a safe and harmonious coexistence between human workers and collaborative robots requires these robots to possess adaptive decision-making abilities, including active collision avoidance and reactive robotic planning. In this pursuit, the accurate recognition of human activities plays a pivotal role in the realm of human–robot collaboration [6,7]. Precise human activity recognition empowers robots with the ability to anticipate and respond to human movement, effectively mitigating the risk of collisions and accidents in dynamic construction environments. Moreover, the ability to precisely recognize and understand human activities allows robots to perceive the context and intentions of human workers, facilitating seamless collaboration and synchronization of actions. As a result, this level of human activity recognition not only optimizes workflow efficiency but also enhances the user experience by fostering smooth interaction and effective communication between humans and robots. It is within this context that accurate human activity recognition becomes the cornerstone of success for the rapidly growing field of human–robot collaboration in construction, paving the way for safer, more efficient, and harmonious work environments.

With the rapid advancement in technology, there has been a growing body of research focused on human activity recognition using motion sensors, lighting sensors, GPS, image sensors, etc. Among these studies, vision-based approaches have gained considerable attention due to their non-intrusive nature and real-time identification of diverse activities [8]. Vision-based human activity recognition, primarily relying on pose detection with the assistance of human skeletons, allows for the extraction of informative features. Human skeletons provide a spatial representation of body configurations, capturing the spatial relationships and dynamics of movements. This spatial information, combined with reduced dimensionality, enhances the efficiency of the recognition process. Three-dimensional estimation of human pose is crucial in this context, as it enables an accurate understanding and interpretation of various human activities [9]. By capturing the spatial and temporal relationships among body joints and segments, 3D pose estimation enables the extraction of significant features that are crucial for discerning between various activities [10,11,12,13]. It aids in identifying subtle variations in body poses and movements, facilitating the discrimination of similar actions or activities with distinct characteristics. Therefore, 3D estimation of human pose is indispensable in human activity recognition, providing a foundation for robust and accurate systems capable of understanding and interpreting human actions in the context of human and robot collaboration in construction.

Numerous studies have investigated 3D human pose estimation using different approaches, including single-camera, RGB-D camera, and multi-camera methods. The single-camera approach is a commonly employed technique used to infer the 3D position and orientation of a human from a 2D project obtained through a single camera [14,15,16,17,18,19]. However, single camera pose estimation suffers from a lack of depth information and occlusion [20]. The RGB-D camera approach can capture both RGB and depth information simultaneously, leading to more accurate 3D reconstruction compared to a single-camera approach [21,22]. Nonetheless, RGB-D cameras also have limitations in terms of depth measurement accuracy at extreme ranges, narrow field of view, and sensitivity to lighting conditions, which can affect the completeness, accuracy and reliability of 3D pose estimation [23]. Researchers have investigated multi-camera pose estimation techniques to overcome the limitations of single-camera and RGB-D camera pose estimation. These techniques, including triangulation and Kalman filtering, present potential solutions [24,25,26,27,28]. However, triangulation heavily relies on precise feature matching and assumes known camera parameters, making it vulnerable to occlusion and complex backgrounds. Additionally, the Kalman filter assumes linearity and Gaussian characteristics, which may not accurately capture the non-linear and non-Gaussian aspects of human motion. All in all, while multi-camera pose estimation holds promise for addressing some of the limitations of single-camera and RGB-D camera approaches, challenges related to feature matching, camera parameters, and accurately modeling human motion dynamics remain significant considerations. These challenges can impact the accuracy and reliability of 3D human pose estimation in complex and dynamic environments, such as human–robot collaboration tasks in construction settings. Moreover, in complex environments, such as human–robot collaboration tasks in construction settings, occlusions can occur frequently, hindering the accurate estimation of 3D human pose. Occlusions can lead to incomplete or ambiguous feature matching, impacting the reliability of traditional pose estimation techniques.

To address these challenges, this study proposes a novel multi-camera-based approach using a particle filter algorithm. The particle filter is employed not only to optimize feature matching but also to effectively handle occlusions and complex backgrounds. Rather than relying solely on precise feature matching, the particle filter incorporates probabilistic models of feature correspondences, facilitating more robust estimation even in demanding scenarios. The study follows a two-step approach to achieve its objectives. Initially, the 2D locations of human body joints are extracted from multiple camera views. To obtain comprehensive 3D joint locations, a fusion technique utilizing the particle filter algorithm is employed, effectively integrating information from various perspectives. Subsequently, a robust deep learning model is designed to accurately classify activities based on the extracted joint locations. This two-step process ensures the efficient integration of multi-view data and enables accurate activity recognition for human–robot collaboration in construction settings. The novel contributions of this study lie in the effective integration of multi-view data through the particle filter algorithm, the accurate activity recognition facilitated by the deep learning model, and the robustness in handling occlusions during 3D pose estimation. These contributions pave the way for safer and more efficient human–robot collaboration in construction, showcasing the potential of this approach to advance the field of construction automation and safety.

The paper is structured as follows. Section 2 provides an overview of the existing techniques for 3D pose estimation and human activity recognition. Section 3 describes the proposed methodology, which includes the estimation of 3D human pose, and the use of LSTM for human activity recognition. In Section 4, the paper presents the data collection experimental design and model implementation. The activity recognition results for both single- and multiple-camera approaches, as well as findings and implications are analyzed and discussed in Section 5, which also includes recommendations for the best approach for activity recognition. Finally, Section 6 provides a summary of the study, its limitations, and suggestions for future research.

## 2. Related Works

Vision-based pose estimation is essential in human activity recognition as it provides information about the body’s joint positions and movements. It involves estimating the spatial locations of various body joints, such as shoulders, elbows, knees, etc. In the context of human–robot collaboration, 3D human pose estimation becomes particularly valuable. By estimating the spatial positions of body joints in three-dimensional space, it enhances the robot’s perception and understanding of human actions. Three-dimensional pose estimation can be achieved through various means, such as using a single camera, RGB-D cameras, or multiple cameras.

Single-camera human pose estimation is a widely used technique that involves reconstructing the 3D position and orientation of a human from a 2D projection captured by a single camera. Due to its simplicity and cost-effectiveness, it is commonly used in human pose estimation tasks. Ref. [14] demonstrated the efficacy of this approach in recovering pose solutions using a single view. Refs. [15,16] introduced an example-based method for 3D pose estimation from single-view image sequences. More recently, ref. [17] proposed a CNN-based approach to real-time 3D pose estimation using a single fisheye lens camera with a wide field of view and egocentric views of a person’s body movements. Refs. [18,19] introduced a technique for estimating full-body pose from monocular images taken by a downward-facing fisheye camera installed on a Head-Mounted Display. Ref. [24] developed a method for estimating globally stable 3D human poses from a single fisheye camera mounted on a head, using 2D and 3D key points detected by a CNN and motion priors based on a variational autoencoder. However, single camera pose estimation has limitations, such as loss of depth information and occlusion. In a single-camera setup, the lack of depth information can make it challenging to recover the 3D pose from a single 2D image. Occlusion occurs when some parts of the body are obstructed or blocked from view, leading to pose estimation errors as the missing coordinates of body joints cannot be inferred from the available data. This encompasses various types of occlusions, such as self-occlusion where different body parts obstruct each other, occlusion by external objects, and partial occlusion of human body parts [29].

An RGB-D camera is capable of capturing RGB and depth information simultaneously, resulting in more accurate 3D reconstruction compared to a single camera setup. The research outlined in [30] builds upon the utilization of the Microsoft Kinect sensor and introduces a novel approach to human activity recognition through the application of machine learning. In another study, a research paper [31] presented an online method for human activity recognition utilizing RGB-D sequences captured by a Kinect device. The method employed a combination of multiple fused features, namely depth silhouettes and human skeletons. Thorough examinations conducted on three standard depth datasets demonstrated that the proposed approach achieved exceptional outcomes, surpassing existing methods in the field. Ref. [32] proposed a 3D human pose and camera calibration tracking approach that utilized three RGB-D Kinect sensors. Ref. [22] used RGB-D videos to generate a parametric 3D deformable human mesh model for extended pose estimation. However, RGB-D cameras have a limited range within which they can accurately measure depth. Objects or body parts that are too far away or too close to the camera may suffer from depth measurement errors. Furthermore, since RGB-D cameras rely on structured light or time-of-flight technology to capture depth information, they can be sensitive to lighting conditions, such as bright or dark environments. Lighting conditions can affect the accuracy and reliability of depth measurements, which in turn can impact the quality of 3D pose estimation. In addition, RGB-D data is characterized by its low resolution, which leads to image noise due to reduced sensitivity. This type of data can be susceptible to interference from certain materials, such as those that are light-absorbing or transparent [33].

Several studies have aimed to overcome the limitations of single-camera and RGB-D camera pose estimation through the exploration of multi-camera-based pose estimation. This approach is more robust than previous methods as it is less susceptible to occlusion and provides more depth information, making it suitable for real-time tracking of subjects in complex environments where the subject may move quickly and unpredictably. Refs. [24,25,34] have proposed methods for multi-camera-based 3D pose estimation that use direct triangulation of 2D joint detections. Ref. [26] have developed a weakly supervised 3D pose estimation approach that combines temporal information and triangulation. They estimate the 3D pose by triangulating the location of body joints in each camera view. The 3D position of a joint is computed using multiple 2D projections of the same 3D point. However, triangulation relies on having complete and accurate measurements from all camera views. In scenarios where some measurements are missing or incomplete, triangulation may struggle to provide reliable pose estimation. Kalman filters can handle missing or incomplete measurements by utilizing a prediction-correction mechanism. They can predict the pose based on the dynamic model even when certain measurements are missing and then update the estimate once the measurements become available. Ref. [27] used Kalman filter to estimate the internal state of a linear dynamic system from a series of noisy measurements for trajectory generation to ensure safe human–robot collaboration. Ref. [28] adopted the extended Kalman filter to estimate the pose using noise covariance matrices based on the sensor output. However, the Kalman filter assumes a linear relationship between the observed data and the hidden state variables, which may not hold true in reality. The relationship between the observed 2D joint positions and the underlying 3D pose is highly nonlinear, which can potentially limit the accuracy of the filter.

To overcome the limitations of the linear assumption in the Kalman filter, this study proposed a particle filter approach, which is a probabilistic technique, for 3D human pose estimation using a multiple-camera system. A particle filter represents the state estimate as a collection of particles, each representing a hypothesis about the hidden state. This allows for the flexible and accurate modeling of complex nonlinear systems. Particle filters offer robust and accurate 3D human pose estimation, making them well-suited for human–robot collaboration in construction tasks. Their ability to handle nonlinearities, occlusions, real-time adaptability, sensor fusion, uncertainty handling, and complex human movements contribute to improved performance and effectiveness in construction scenarios [35,36,37].

In the field of 3D skeleton-based human activity recognition, researchers have explored two main approaches: hand-crafted feature-based methods and deep network-based methods. Unlike hand-crafted feature-based methods, deep network-based methods leverage the ability to directly learn features from the data for human activity classification. In recent years, deep learning-based methods, particularly LSTM networks, have demonstrated exceptional performance in accurately recognizing and understanding human activities in the context of human–robot collaboration scenarios. LSTM networks are a type of recurrent neural network (RNN) specifically designed to model sequences of data [38]. By analyzing the sequence of 3D pose estimates, LSTM networks can learn the patterns and transitions between different poses, enabling accurate recognition of the activity being performed. Additionally, LSTM networks are capable of handling sequences of varying lengths, which is crucial in human activity recognition since the duration of activities can vary significantly. Due to these reasons, researchers have proposed LSTM networks as a more sophisticated approach to capturing the nuances of human actions and various studies have highlighted their effectiveness [39,40,41]. As such, LSTM networks offer a promising approach for accurately recognizing human activities, enhancing the understanding of complex movements observed in construction work, where comprehending the sequence of actions performed by a worker is essential for understanding the overall workflow and ensuring worker safety. Therefore, in this study, LSTM networks were employed to identify human activities based on the 3D joint locations of the human body. By understanding the patterns and transitions among different poses, LSTM networks facilitate precise classification of diverse human actions, even in scenarios characterized by unpredictable and imbalanced motion distributions.

It is also worth exploring methodologies from other fields that have demonstrated success in target location and detection. For instance, hyperspectral images provide wealthy spectral information, but processing high-dimensional data with redundant bands poses challenges. Band selection (BS) addresses this issue by reducing redundancy and focusing on relevant bands [42]. Additionally, super-resolution mapping (SRM) based on spatial–spectral correlation enhances mapping results [43]. Adopting insights from spectral imagery and target detection fields can advance human activity recognition in construction settings.

## 3. Methodology

In this study, a novel approach is proposed to recognize human activities using a particle filter in a multiple-camera setting. The study involves a two-step process. Firstly, 2D location of human body joints are extracted from the camera views. To combine information from different views and generate 3D joint locations, a fusion method employing a particle filter is applied. In the second step, a deep learning model is developed to classify the activities based on the joint locations. LSTM networks are trained using labeled datasets containing sequences of 3D joint locations corresponding to various human activities, particularly those related to human–robot collaborative tasks in construction. The trained LSTM model is then utilized to recognize human activities.

### 3.1. Estimating 3D Human Pose Using Particle Filter

**Figure 1:**
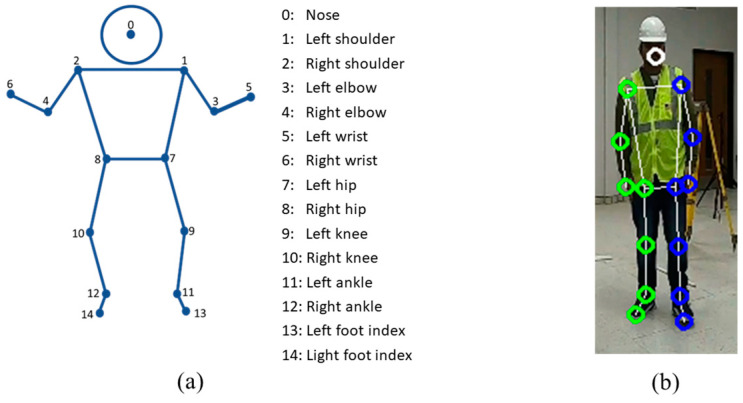
MediaPipe pose detector: (**a**) all defined human 2D joint locations; (**b**) 2D joint locations for extracted for the experiment.

An existing human pose estimation technique is used here to extract the 2D joint locations from an image obtained from a camera. Because the performance of the 3D pose estimation process depends on the base 2D joint detection technique, the algorithm should have high accuracy and real-time performance to be effective. In this research, a state-of-the-art technology called MediaPipe Pose was utilized because it is a pre-trained model and a standardized pipeline for processing video frames or images. MediaPipe Pose is one of the cutting-edge technologies that has the capacity for high-fidelity pose identification and real-time operation. To predict joint locations in relation to the image coordinates, MediaPipe Pose uses a two-step detector–tracker technique that first finds the pose region-of-interest (ROI) in the image [44]. The 2D coordinates of 15 main key points out of 33 landmarks are extracted here from four cameras to be fused for pose estimation. Figure 1 shows the MediaPipe application for joint detection.

The results of the 2D estimation are considered noisy observation and are used as inputs by the probabilistic estimation algorithm because the 2D pose estimation algorithm frequently fails to detect the human pose or generates the wrong pose estimate due to self-occlusion or occlusions by any object or other bodies. Indeed, since the real world is in three dimensions, using images that contain two dimensions results in losing one important part of the information. Hence, it is necessary to generate 3D coordinates using images taken from different viewpoints.

To combine the data obtained from the multiple cameras and obtain a 3D estimation of the human pose, the particle filter technique is employed. Particle filter is an algorithm for estimating unknown internal states based on partial and noisy observations. Particle filter has several advantages in multi-camera pose estimation. The particle filter algorithm can handle non-linear and non-Gaussian distributions of data, which is commonly encountered in multi-camera pose estimation. It also can handle missing or corrupted data, which can occur due to occlusions or other factors in multi-camera environments. Particle filters are computationally efficient and can be used in real-time applications. In addition, it can achieve high accuracy in multi-camera pose estimation by fusing information from multiple cameras and taking into account the uncertainties in the data. Hence, this study employs the particle filter algorithm to estimate 3D human pose with the aim of exploiting its advantages.

In this research, a particle represents a 3D human pose consisting of the three-dimensional location of 14 joints. Given the camera’s location and orientation, the joints within each particle are projected onto the camera plane, producing expected locations on the camera’s image. These projected joint locations serve as expectations. Meanwhile, the actual joint locations on the captured camera image are detected using a human pose detector. These detected locations are considered noisy observations due to potential errors caused by factors like occlusions or lighting conditions. The error between the expectations and observations for each joint is defined as the distance, and it is assumed that the error follows a normal distribution with a mean of 0. To find the most likely human pose that corresponds to the observed data, the particle filter algorithm iteratively calculates the likelihood and performs resampling of the particles. The estimation with the highest likelihood represents the human pose that is deemed most probable given the observations.

In Algorithm 1, an illustration of the particle filter algorithm is shown used for the estimation process. It begins with a set of randomly distributed particles (Line 1). Because it is impossible to predict how humans move, the pose is modeled by a free-rolling ball at each joint that moves in a random direction (Line 7). After that, the human pose in each particle is projected to an image plane (Line 11) as if it were caught with the i-th camera, and the projected joint locations are compared to the observations (Line 12). The Euclidean distance between two locations is considered a Gaussian random variable and is used to update the weight of each particle. This is carried out for each pair of expected and observed joint locations on the image coordinate (Line 13). After that, the weights of the particles are normalized (Line 16), and another sampling of the particles is done based on their weights (Line 17). To provide the posterior distribution of the 3D human position, the particles are iteratively updated such that they reflect the fact that a particle with a high weight has a higher probability of being resampled.
**Algorithm 1** Pseudocode for the particle filter algorithm used in 3D human pose estimation—EstimatePose(N, M, C)**Parameters**   N: number of iterations   M: number of particles   C: Set of location and orientation of camera sensors1:     P = initialize_particle(M) // A particle has 3D locations (x_i_, y_i_, z_i_) of i-th joint for I = 0,…,14 2:     **for** n = 1 to N **do** 3:        **for** i = 1 to n(C) **do** 4:           image[i] = capture_frame(C[i]) 5:        **end for** 6:        **for** m = 1 to M **do** 7:           P[m] += N(0, Σ) // Pose change as a free rolling ball 8:           W[m] = 0 9:           **for** i = 1 to n(C) **do** 10:            // Expectation and observation of 2D locations of joints on the image coord. 11:            expected_m,i_ = project_2d(P[m], C[i]) 12:            observation_i_ = detect_pose_2d(image) 13:            W[m] = update(W[m], expected_m,i_, observation_i_) 14:         **end for** 15:      **end for**
16:      W = normalize(W) 17:      P = resample(P, W) 18:   **end for** 19:   Return estimated pose

### 3.2. Recognizing Human Activities Using LSTM with 3D Joint Locations

This research utilizes the LSTM model to identify human activities, as it excels in identifying patterns within time series data [45]. The selection of LSTM networks for activity recognition is based on their inherent ability to model sequential data and retain long-term dependencies. LSTM, as a specialized type of RNN model, possesses improved capabilities in computing hidden states. In contrast to alternative RNN approaches, LSTM has shown remarkable proficiency in activity recognition and classification and predicting trajectories [46].

LSTM networks are designed with multiple gates that allow the network to selectively remember or forget information at each time step. The input gate of an LSTM unit discovers which value from input should be used to modify the memory as shown in Equation (1). Sigmoid function maps values to the range of 0 to 1. and the tanh function gives weightage to the values which are passed deciding their level of importance ranging from −1 to 1, expressed in Equation (2).

*i_t_* = *σ* (*W_i_* * [*h_t_*_−1_, *x_t_*] + *b_i_*) (1)

*c_t_* = tanh(*W_c_* * [*h_t_*_−1_, *x_t_*] + *b_c_*) (2)

The forget gate discovers what details are to be discarded from the block as shown in Equation (3). It is decided by the sigmoid function. It looks at the previous state (*h_t_*_−1_) and the content input (*x_t_*) and outputs a number between 0 (omit this) and 1 (keep this) for each number in the cell state *C_t_*_−1_.

*f_t_* = *σ* (*W_f_* * [*h*_(*t−*1)_, *x_t_*] + *b_f_*) (3)

**Figure 2:**
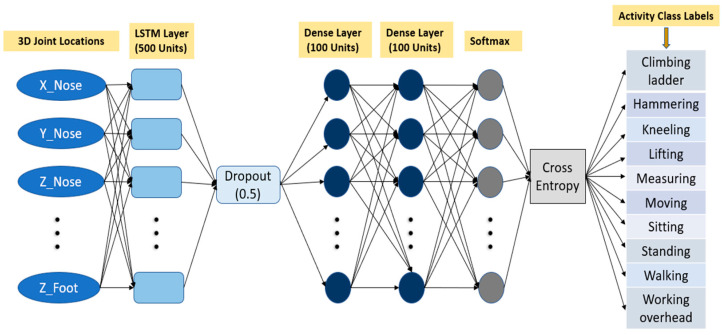
Network architecture of LSTM model for human activity recognition using 3D joints location.

In the output gate, the input and the memory of the block are used to decide the output, expressed in Equation (4). Sigmoid function restricts the output values between 0 and 1. and the tanh function gives weightage to the values which are passed deciding their level of importance ranging from −1 to 1 and multiplied with output of Sigmoid as shown in Equation (5). The detailed structure of our model is illustrated in Figure 2.

*o_t_* = *σ* (*W_o_* * [*h*_(*t−*1)_, *x_t_*] + *b_o_*) (4)

*h_t_* = *o_t_* * tanh(*C_t_*) (5)

The process of hyper-parameter optimization, a critical step in fine-tuning the performance of the model, was meticulously conducted using KerasTuner, an indispensable and powerful tool within the Keras library. This approach allowed us to systematically explore and select the optimal hyper-parameters, ensuring that the final network comprises the most effective configuration. As a result of this process, the ultimate architecture of the designed network architecture integrates the following chosen components, each contributing to its overall robustness and accuracy. An LSTM layer of 500 units, followed by a dropout of 0.5 and two dense layers with size 100. The rectifier linear unit (ReLU) activation function is used in all hidden layer units. It is common to increase the number of nodes in the network if the input sequence has many inputs. The reason for this is that increasing the number of nodes allows the network to learn more complex patterns and relationships in the input data. This is particularly important when dealing with high-dimensional input data such as images, videos, and sensor data. However, this also increases the risk of overfitting the data, which occurs when the network becomes too specialized to the training data and performs poorly on unseen data. The dropout layer prevents individual neurons from becoming too specialized and encourages the network to learn more generalizable features. This helps to reduce overfitting and improve the model’s ability to generalize to new data. Moreover, dropout can also be used to reduce the computational cost of training large LSTM models by randomly dropping out some of the neurons and thus reducing the number of computations required during training. This can speed up training time and reduce memory requirements. Since the activity recognition is a classification problem, the SoftMax activation function is used at the end. The model is then optimized using the Adaptive Moment Estimation (Adam) optimizer and “categorical cross-entropy” loss function. The SoftMax activation function is commonly used in the output layer of a LSTM network to convert the output of the last layer into a probability distribution over the possible output classes, and the categorical cross-entropy loss is then calculated by comparing the predicted probabilities with the true labels. The coordinates information of 15 key points of the body from a past window period will be fed to the model as an input vector, and the expected output is the probable class of activity performing in corresponding frame.

## 4. Experiments

### 4.1. Data Collection and Model Implementation

Several studies have focused on classifying construction workers’ activities. Ref. [47] identified workers’ activities such as preparing material, placing material, consolidating material, and plastering walls. Ref. [41] conducted the automatic recognition of workers’ motions in highway construction including standing, walking, bending-up, bending-down, twisting, and working overhead. Ref. [48] used smartphone-based technology to recognize activities such as sawing, hammering, turning a wrench, loading, hauling, unloading, and returning. However, these previous activities did not cover human–robot collaborative tasks that required working alongside existing construction robots.

To identify human activities associated with human–robot collaborative tasks in construction, this study examined existing construction robots, their functions, and the behaviors of collaborative human workers, as shown in Table 1. Although construction robots can perform several tasks with high accuracy, they still need the assistance of human co-workers to achieve a proper finish or ensure work quality. To collaborate effectively with construction robots, certain human behaviors are necessary such as accurate placement, cleaning up mortar, excavating with a rake, measuring with a tape, and supervision, etc. Based on their collaborative behaviors, this study identified a total of ten activities of human workers in conjunction with construction robots. These activities include standing, sitting, walking, lifting objects, moving objects, kneeling, hammering, measuring, climbing ladders, and working overhead.

**Figure 3:**
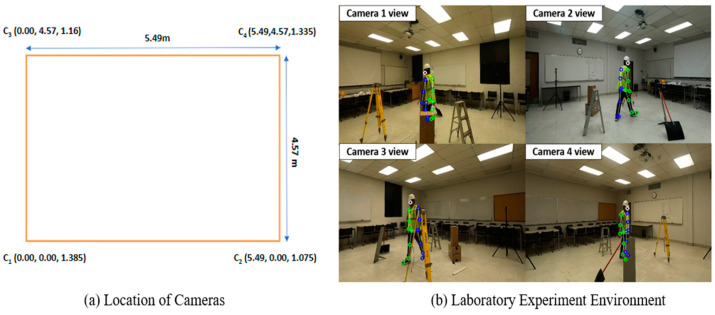
Laboratory experimental settings.

To prepare the training datasets for the activity recognition model, an experiment capturing videos of a human subject while performing ten different construction activities was carried out in the laboratory environment. The laboratory space measured 5.49 (l) meters by 4.57 (w) meters by 2.59 (h) meters, as shown in Figure 3. Four GoPro HERO8 Black cameras were strategically placed to capture 1080p high-definition footage of the human subject’s movements from multiple angles. The cameras’ horizontal field of vision (FOV) was set to 118.2 degrees, and the vertical field of view was set to 69.5 degrees when the linear lens was selected as the camera’s digital lens mode. To enable the multi-camera system to simultaneously record videos, capturing each frame from different views concurrently, four cameras need to be synchronized. In the data collection process, the participant was instructed to produce a distinct and audible clap sound at the beginning and end of the recording session. This simple yet effective technique served as a synchronization marker across all camera angles. Using Adobe Premiere Pro (version 23.4), a professional video editing software, a Multicamera Sequence was created, and the clap sounds were utilized as synchronization points. This allowed us to precisely align the video clips from different camera angles during the post-production phase. As a result, the start and end of all the different view videos were accurately aligned, facilitating seamless and coherent further analysis.

**Figure 4:**
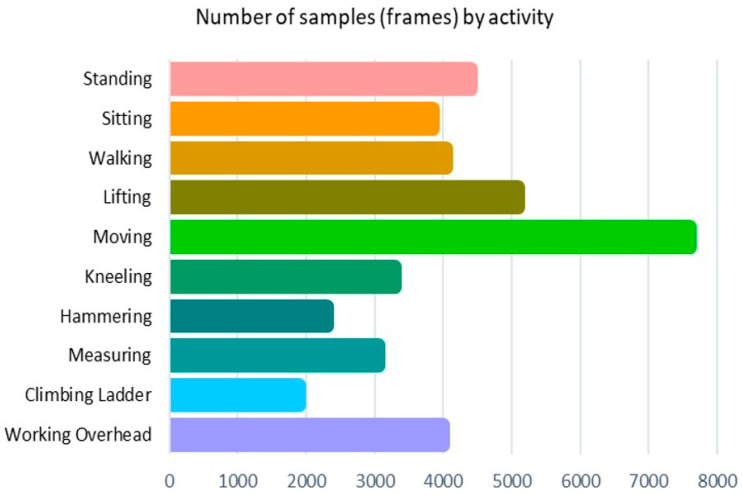
Number of frames captured for each activity.

The experiment involved collecting data on a human subject performing various human–robot collaboration tasks, including standing, sitting, walking, lifting objects, moving objects, kneeling, hammering, measuring, climbing ladders, and working overhead. The videos were captured at a rate of 30 frames per second, with a total length of 22 min and 12 s. After extracting the joint locations of each frame, the resulting dataset contained 15 sets of 3D coordinates, along with their corresponding activity classes. Figure 4 illustrates that moving objects had the highest-class label distribution in the dataset, while climbing ladders had the lowest.

To ensure the robustness and applicability of the proposed method in real-world scenarios, it is essential to consider various factors that may influence the performance of the equipment. External variables, such as surrounding temperature, lighting conditions, and environmental complexity, can significantly impact the accuracy and reliability of the activity recognition system. Temperature fluctuations, for example, may affect the performance of sensors and cameras, leading to potential variations in data quality. Additionally, complex surroundings with cluttered backgrounds or occlusions could pose challenges for accurate human pose estimation. While conducting experiments under diverse environmental conditions would provide valuable insights, the scope of the current study was limited to a controlled environment.

The LSTM model requires fixed-length sequences as training data. Therefore, the dataset was segmented, and each generated sequence (or window) contains 100 records corresponding to 1.66 s. To ensure continuity between activities, overlapping windows with an 80% overlap were considered. The dataset used to train the human activity recognition model consisted of 45 coordinates of 15 joints, which are the model features, and one activity label, which is the target variable. To evaluate the model’s performance, the data was split into 65% for training, 15% for testing, and 20% for validation. This step is crucial in developing accurate and reliable machine learning models. The model was trained for 100 epochs using a learning rate of 0.0002 on both the training and validation data. The experiments were conducted on a computer with an Intel(R) Core (TM) i7-1195G7 CPU running at 2.90 GHz, 16 GB RAM, a 64-bit operating system, Windows 11 Home, and Intel iRIS Xe graphics, using Python 3.10.

### 4.2. Performance Measurement

The confusion matrix was used to evaluate the performance of the developed models. It displays the number of instances where the predicted activity matches the actual activity and where it does not, as shown in Table 2. The rows of the matrix represent the actual activities, while the columns represent the predicted activities. The diagonal elements indicate the number of correctly recognized activities, while the off-diagonal elements represent the misclassified activities.

Performance metrics, including accuracy, precision, recall, and F1-score, were also employed to evaluate the performance of the developed models. Accuracy is a commonly used performance measure that evaluates how well a model predicts the target variable. Recall is a metric that measures the ratio of true positive instances to all actual positive instances. Essentially, it assesses the model’s ability to accurately identify all instances of a particular activity. Precision, on the other hand, measures the ratio of true positive instances to all instances that the model predicted as positive. It reflects the model’s ability to avoid mistakenly recognizing an activity that is not happening. The F-score is a composite measure that balances the trade-off between recall and precision. It is calculated as the harmonic mean of recall and precision and ranges from 0 to 1, with 1 indicating optimal performance. These metrics help to measure the effectiveness of the models in predicting the target variable. The calculation process for each evaluation metric is demonstrated in Equations (6)–(9).

Accuracy = (TN + TP)/(TP + TN +FP + FN)(6)

Precision = TP/(TP +FP)(7)

Recall = TP/(TP + FN)(8)

F1-score = 2 × (Precision × Recall)/Precision + Recall(9)

In addition, this study used the Precision–Recall (PR) curve to analyze the trade-off between precision and recall for various activities. The curve was plotted by calculating precision and recall values at each threshold and plotting the resulting points. A high Area Under the Curve (AUC) for the PR curve indicates that the model is achieving high precision and recall values across a range of decision thresholds, which suggests that the model is effective in recognizing activities with minimal false positives or false negatives. Conversely, a low AUC for the PR curve suggests that the model may have low precision, low recall, or both, indicating poor performance. In this study, the performance of the proposed approach has been evaluated and compared for one, two, three, and four cameras, and the results were compared for each performance metric.

## 5. Results and Discussion

### 5.1. Experimental Results

**Figure 5:**
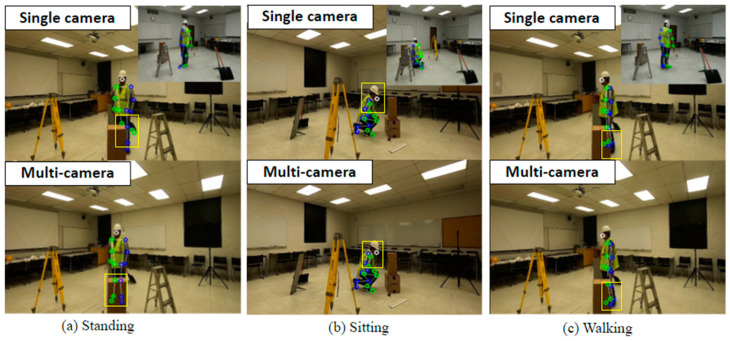
Comparison of 3D pose estimation results. **Top**: using a single camera; **bottom**: using multiple cameras.

**Figure 6:**
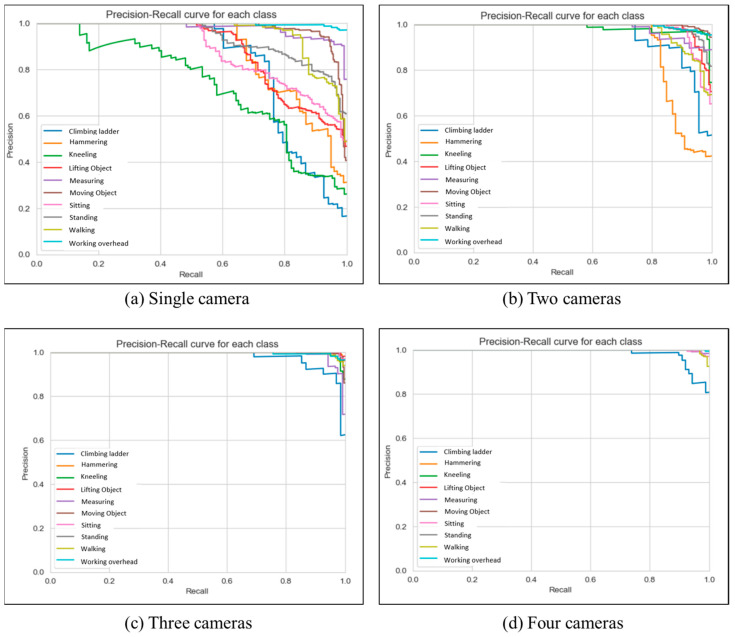
Precision–recall curve for the LSTM classification model.

Figure 5 presents a comparison of the results of 3D pose estimation using a single camera versus multiple cameras. When only a single camera was employed, several lower body key points could not be correctly identified and estimated when it comes to occlusions. On the other hand, the proposed method could successfully estimate the occluded key points from the multi-views (see Figure 6a). In the scenario in which the human subject was sitting, a single camera view was unable to correctly estimate the joint locations of the left-side body points, but the multiple camera approach correctly identified them (see Figure 6b). When a human was walking, the right-side key points and the ankles and feet could not be detected with a single camera, whereas the suggested approach accurately detected all joint positions by fusing the information collected from the rest of the cameras (see Figure 6c).

**Figure 7:**
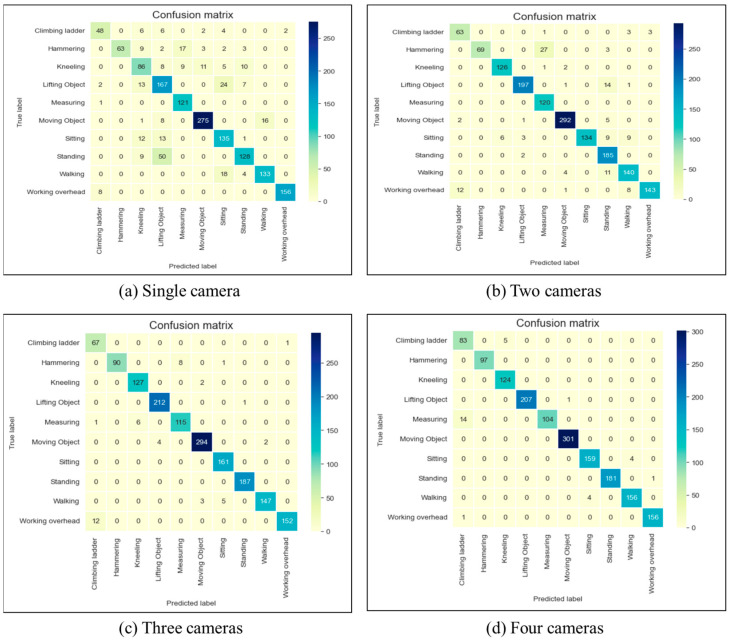
Confusion matrix for the LSTM classification model.

This study aims to evaluate the feasibility of utilizing a multi-camera setup with a particle filter to accurately recognize human activities. Therefore, the performance of human activity recognition models using one, two, three, and four cameras were compared. In single-camera-based models, the 2D locations of joints are utilized. The performance metrics, precision-recall curves, and confusion matrixes for each activity are shown in Table 3, Figure 6 and Figure 7, respectively.

The accuracy of the activity recognition through a single camera was found to be 82% and the precision, recall and F-1 score are lower for most of the activity classes compared to the multiple camera performance. The model demonstrated high precision (i.e., generated fewer false alarms) for hammering, moving, and working overhead. Additionally, the model exhibited higher recall (i.e., accurately recognized the activities) for moving, measuring, and working overhead.

When using two cameras, the recognition of activities yielded a 92% accuracy, indicating a noteworthy advancement that ensued by transitioning from 2D data to 3D. The precision, recall, and F-1 score for the majority of activities were superior when compared to the single camera’s performance, indicating that using two cameras leads to better identification of activities and minimizes the possibility of falsely recognizing them. For example, when it came to hammering, kneeling, lifting objects, moving objects, sitting, and working overhead, the model achieved a precision close to 1.0. Similarly, the model had higher recall values for climbing ladder, kneeling, lifting object, moving object, measuring, standing, walking, and working overhead. These results denote the model’s proficiency in preventing erroneous positive forecasts for these activities, even in circumstances where there is occlusion or inadequate visibility. In light of the findings, it can be ascertained that the implementation of a dual-camera system generally enhances the recognition performance across a wide range of scenarios, thereby offering a more accurate and reliable means of identifying and differentiating between various activities.

Using the dataset from three and four cameras, the model achieved an accuracy rate of 97% and 98%, respectively, representing significant progress compared to the single-camera (15%) and the two-camera approach (5%). Also, precision, recall and F1-score increased significantly for most activities compared with the two-camera approach. For all the activities except climbing ladder and measuring, both models gained a precision of more than 0.95, indicating its ability to avoid generating false alarms of these activities. Also, it is evident from the precision–recall curve that the three- and four-camera approaches have an area under their curves that is noticeably larger than the other two. On the other hand, although the AUC of four-camera system is greater than three-camera, other performance measures are nearly the same with only minor differences in a number of tasks, some in favor of a four-camera approach and some in the other direction. Overall, these findings suggest that using multiple cameras generally enhances the recognition performance, providing a more accurate and reliable means of identifying and differentiating between various activities.

### 5.2. Discussions

**Figure 8:**
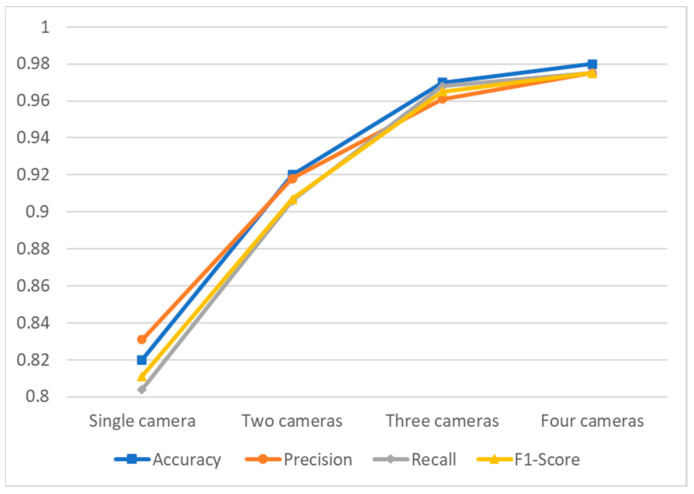
Overall performance metrics comparison.

This study proposes a human activity recognition model using a multiple camera system, incorporating particle filter and LSTM. To evaluate the performance, the proposed model was tested in four different setups using one to four cameras. The results show that models with high recall and low precision can recognize many activities but generate false alarms, while models with high precision and low recall miss many activities. Thus, a human activity recognition model requires high recall and precision values to be accurate, and a high F1 score is necessary to achieve a good balance between precision and recall. Figure 8 illustrates that the accuracy, precision, recall, and F1-score increase with the increase in number of cameras.

The single-camera setup had to deal with occlusions and a lack of visibility, resulting in lower accuracy and performance metrics. However, the two-camera setup significantly improved the model’s performance, and the three-camera setup showed even better recognition activity due to better visibility. The precision-recall curves also indicate that the areas under the curve (AUC) of all activity classes increase with the number of cameras, indicating that the model can recognize activities more accurately without producing many false positives or false negatives. Overall, the three- and four-camera approach demonstrated the highest accuracy, precision, recall, and F1 score in recognizing activity using the LSTM model, even when the human subject is not facing the camera, or some body parts are occluded by objects in a construction environment. The utilization of multiple cameras in experimental setup allowed us to capture human actions from different angles, providing comprehensive data for 3D human pose estimation. This approach yields potential advantages, such as improved accuracy in pose estimation and enhanced activity recognition performance. By fusing information from multiple cameras, the system can account for occlusions and obtain a more complete representation of human movements. Moreover, a multi-camera setup facilitates the generation of 3D joint coordinates, which are crucial for precise activity recognition.

The proposed multi-camera-based approach offers several advantages, including enhanced recognition performance and accuracy in 3D human pose estimation and activity recognition. However, it is crucial to recognize the trade-offs associated with this method, considering both computational cost and the cost implications of utilizing multiple cameras.

Utilizing multiple cameras for data fusion and pose estimation may increase the computational burden compared to single-camera or RGB-D camera approaches. The proposed approach involves several critical components that contribute to the computational cost. Firstly, the extraction of 2D joint locations from multiple camera views incurs a certain time overhead. Additionally, the fusion method employing the particle filter algorithm for generating 3D joint locations introduces further computational complexity, which scales with the number of cameras used. The integration and synchronization of data from different viewpoints require significant processing resources. Furthermore, the subsequent deep learning model, specifically the LSTM network, contributes to the overall time complexity during the activity recognition phase. The LSTM processes sequential data and may require extensive computations for accurate classification of human activities.

By analyzing the time complexity and cost implications of each component, it becomes evident which aspects of the proposed method have the most substantial impact on performance. This insight is valuable for identifying potential areas of optimization and making informed decisions regarding the deployment of the approach in real-world scenarios. In the experiments conducted in the present study, the time needed for data fusion using four cameras was measured to be 2.62 times higher than that of using two cameras and 1.48 times higher than that of using three cameras. While using a more powerful computer system may reduce these times, it is important to note that the computational cost also translates to actual financial costs. Adding more cameras to the setup may improve accuracy and robustness, but it also incurs higher hardware and maintenance expenses. Thus, a cost trade-off must be carefully considered when deciding the number of cameras to use in a specific application.

The benefits of improved accuracy and robustness achieved through more cameras in the multi-camera setup come with the cost of increased computational resources and financial investment. In real-time applications, where prompt and efficient processing is essential, the additional computational load and cost implications may become critical factors in the decision-making process. Therefore, a comprehensive analysis of both computational cost and cost trade-off is warranted and careful consideration should be given to strike a balance between the number of devices and the desired level of accuracy and system complexity.

By capturing human actions from multiple camera viewpoints, the proposed system comprehensively analyzed movements, allowing for a more complete representation of human actions in collaborative scenarios. The results demonstrated that this approach achieved a successful balance between accurately recognizing activities and minimizing false alarms. This is crucial for promoting safe and harmonious human–robot teamwork, reducing the risk of collisions and injuries. Overall, our findings emphasize the effectiveness of proposed approach in enhancing collaboration between human workers and robots in construction settings, thus contributing to safer and more efficient work environments. Furthermore, the proposed approach can be also applied to other domains beyond construction environments, helping to improve safety and efficiency in close-proximity human–robot collaboration in various industries.

In this study, we applied the particle filter algorithm to estimate 3D human pose from multiple camera views, given its strengths in handling non-linear and non-Gaussian systems. The particle filter proved to be well-suited for our specific task, as it effectively managed the complexities of data fusion and uncertainties involved in 3D pose estimation from different camera angles. Although a direct comparison with other filtering methods, such as the Kalman filter, was not conducted, the research aimed to demonstrate the successful application of the particle filter for determined objectives. While the particle filter exhibited promising results for 3D pose estimation, it is essential to recognize that various filtering methods have their strengths and limitations. Future studies could delve into comparative analyses to explore the performance of different filtering techniques for similar applications. Overall, findings of this study underscore the efficacy of the particle filter in achieving accurate 3D pose estimation from multiple camera views, contributing to advancements in the field of human pose estimation and multi-camera data fusion.

Despite differences in the dataset and defined activities compared to other experiments, our results are comparable to previous studies focusing on vision-based HAR. For instance, Putra et al. [54] achieved significant success in HAR by utilizing multi-view sequences of raw images and obtaining an impressive 90.3% accuracy. Similarly, Siddiqi et al. [55] proposed an innovative Maximum Entropy Markov Model (MEMM) incorporating depth cameras, which led to an outstanding recognition accuracy of 96.3%. While these results are commendable, it is worth noting that depth cameras represent a recent technological advancement that provides 3D data, enabling them to capture additional spatial information. In contrast, our present study achieves comparable performance using regular 2D cameras, which are more widely available and cost-effective. This finding highlights the effectiveness of our method in achieving competitive recognition accuracy without the need for specialized hardware.

Moreover, our proposed vision-based HAR approach can be compared to studies that utilize sensor-based data. Agarwal and Alam [56] introduced a lightweight LSTM-RNN model, achieving an accuracy of 95.78% in predicting human activities. In a separate study, Antwi-Afari et al. [57] demonstrated impressive results, reporting an accuracy of 97.99% for an LSTM network using wearable insole sensors to recognize and classify various uncomfortable working postures. These sensor-based methods have shown promising outcomes. However, one significant advantage of vision-based HAR over sensor-based alternatives is its practicality in real-world settings, particularly in construction sites. Many construction sites are equipped with surveillance cameras for monitoring safety and security. Leveraging these existing camera systems for HAR eliminates the need for additional equipment purchases and installations.

Although this study has the potential to contribute significantly to ensuring safe human–robot collaboration, there are some limitations to its practical use. The limitations include the dependency of the LSTM model on a large dataset for training to achieve higher accuracy, the need to explore the proposed model’s performance in recognizing more activities performed by multiple individuals simultaneously, the reliance on a specific camera type, and the influence of camera availability and placement on the accuracy, which may be limited in some areas of the construction site. Moreover, one important aspect is the incorporation of ablation experiments to perform a systematic analysis of the method’s individual components’ impact on the overall performance. Focusing on ablation experiments, future studies could aim at understanding the significance of each module within the approach. Finally, this study was conducted in a controlled laboratory environment with limited activities. Therefore, there is a future scope of work to be carried out in real-world construction sites with diverse activities to further validate the proposed approach.

## 6. Conclusions

This study presented a novel approach to accurately recognize construction activities of a worker in close-proximity human robot collaboration using multiple cameras. The proposed approach used a particle filter algorithm to fuse the 2D joint locations of a human extracted from multiple viewpoints to estimate the 3D human pose. Using the results of the estimated 3D human pose, this study recognized ten human activities associated with human and robot collaborative tasks in construction with the LSTM network. The results demonstrated that a single-camera approach could accurately recognize activity only when the subject was facing the camera. The single-camera approach often failed when the subject turns left or right or is partially occluded. On the other hand, as the multiple-camera approach could track the joints of the human body even when some of them were not visible from a particular camera, it resulted in more accurate pose estimation and activity recognition. The experimental results showed that the proposed approach achieved higher performance in activity recognition as the number of cameras used increased. The results of this study contribute to detecting specific human activities what might be used to identify any unsafe behavior, such as workers standing too close to a construction robot or a moving machinery or working close to a robot. More accurate poses with location information and activity recognition with more accuracy will aid in predicting the future movement of a worker so that the robot can calculate a safe plan to avoid any collision.

This multi-camera-based approach offers several distinct advantages, including enhanced recognition performance and accuracy in 3D human pose estimation and activity recognition. By integrating information from multiple camera views, the method effectively manages the complexities of data fusion and uncertainties involved in 3D pose estimation, leading to reliable results even in challenging scenarios with occlusions. Furthermore, the vision-based approach demonstrates its practicality and cost-effectiveness by leveraging widely available 2D cameras, making it readily applicable to real-world construction sites equipped with surveillance cameras. The proposed approach can be useful in construction and for any environment where collaborative work between humans and robots is necessary.

Nevertheless, there is still room for enhancing the adoption of the proposed approach in real-world settings. The current study was limited to laboratory experiments conducted in an enclosed environment with a single individual, rather than in a real construction site with multiple workers. Additionally, this study only tracked a limited number of poses of a human subject, while there are likely to be a wide range of poses among construction workers. Future research should focus on tracking the 3D human pose of multiple construction workers with varying poses in a real construction site environment.

## Figures and Tables

**Table 1:** Existing construction robots and required human–robot collaborative activities.

Manufacturer	Construction Robot	Functions	Collaborative Human Workers’ Behaviors	Human Workers’ Activities
Construction Robotics	Semi-Automated Masonry System (SAM) [49]	Lifting the brick, applying mortar, and placing each brick in place	Accurate placement of the bricks, cleaning up excess mortar, and overseeing the overall project	Lifting/Moving/Standing/Walking
Construction Robotics	Material Unit Lift Enhancer (MULE)	lifting and placing heavy material	Ensuring the accurate placement of the materials	Lifting/Moving
Fastbrick Robotics	Hardrian X [50]	bricklaying	accurate placement of the bricks, cleaning up excess mortar	Lifting/Moving
Dusty Robotics	FieldPrinter/Theometrics	Layout and measurement task	Measuring with tape to ensure the accuracy	Measuring
Doxel	Doxel AI [51]	Monitoring job progress	Supervision	Standing/Walking
Advanced Construction Robotics, Inc.	TyBot [52]	Rebar tying	Supervision	Standing/Walking/Sitting/Kneeling
Eternal Robotics	Myro [53]	Wall painting	Supervision	Standing/walking/climbing ladder/Working overhead

**Table 2:** Confusion matrix.

		Actual Class	
		Positive	Negative
Predicted class	Positive	True positive (TP)	False positive (FP)
	Negative	False negative (FN)	True negative (TN)

**Table 3:** Performance metrics comparison for each activity.

No. Camera	Single Camera			Two Cameras			Three Cameras			Four Cameras		
Activity	P	R	F	P	R	F	P	R	F	P	R	F
Climbing ladder	0.81	0.71	0.76	0.82	0.90	0.86	0.84	0.99	0.91	0.85	0.94	0.89
Hammering	1.00	0.64	0.78	1.00	0.70	0.82	1.00	0.91	0.95	1.00	1.00	1.00
Kneeling	0.63	0.67	0.65	0.95	0.98	0.97	0.95	0.98	0.97	0.96	1.00	0.98
Lifting object	0.66	0.78	0.72	0.97	0.92	0.95	0.98	1.00	0.99	1.00	1.00	1.00
Measuring	0.82	0.99	0.90	0.81	1.00	0.89	0.93	0.94	0.94	1.00	0.88	0.94
Moving object	0.95	0.92	0.93	0.97	0.97	0.97	0.98	0.98	0.98	1.00	1.00	1.00
Sitting	0.72	0.84	0.77	1.00	0.83	0.91	0.96	1.00	0.98	0.98	0.98	0.98
Standing	0.84	0.68	0.75	0.81	0.99	0.89	0.99	1.00	1.00	1.00	0.99	1.00
Walking	0.89	0.86	0.88	0.87	0.90	0.89	0.99	0.95	0.97	0.97	0.97	0.97
Working Overhead	0.99	0.95	0.97	0.98	0.87	0.92	0.99	0.93	0.96	0.99	0.99	0.99

P: precision, R: recall, and F: F1-score.

## Data Availability

The data sets during and/or analyzed during the current study are available from the corresponding author upon reasonable request.

## References

[1] Maeda J., Takada H., Abe Y., Maeda J., Takada H., Abe Y. Applicable Possibility Studies on a Humanoid Robot to Cooperative Work on Construction Site with a Human Worker. Proceedings of the 21st International Symposium on Automation and Robotics in Construction.

[2] Bock T., Bulgalow A., Ashida S. Façade Cleaning Robot for the Skyscraper. Proceedings of the 19th International Symposium on Automation and Robotics in Construction (ISARC).

[3] Zhu A., Pauwels P., De Vries B. (2021). Smart Component-Oriented Method of Construction Robot Coordination for Prefabricated Housing. Autom. Constr..

[4] Zhang M., Xu R., Wu H., Pan J., Luo X. (2023). Human–Robot Collaboration for on-Site Construction. Autom. Constr..

[5] Brosque C., Galbally E., Khatib O., Fischer M. Human-Robot Collaboration in Construction: Opportunities and Challenges. Proceedings of the HORA 2020—2nd International Congress on Human-Computer Interaction, Optimization and Robotic Applications.

[6] Lee S.U., Hofmann A., Williams B. A Model-Based Human Activity Recognition for Human-Robot Collaboration. Proceedings of the 2019 IEEE/RSJ International Conference on Intelligent Robots and Systems (IROS).

[7] Roitberg A., Perzylo A., Somani N., Giuliani M., Rickert M., Knoll A. Human Activity Recognition in the Context of Industrial Human-Robot Interaction. Proceedings of the 2014 Asia-Pacific Signal and Information Processing Association Annual Summit and Conference, APSIPA 2014.

[8] Agrawal S.C., Tripathi R.K., Jalal A.S. (2021). Human Fall Detection Using Video Surveillance. ACS J. Sci. Eng..

[9] Gupta A., Gupta K., Gupta K., Gupta K.O. Human Activity Recognition Using Pose Estimation and Machine Learning Algorithm. Proceedings of the ISIC’21: International Semantic Intelligence Conference.

[10] Nguyen H.C., Nguyen T.H., Scherer R., Le V.H. (2023). Deep Learning for Human Activity Recognition on 3D Human Skeleton: Survey and Comparative Study. Sensors.

[11] Xing Y., Zhu J. (2021). Deep Learning-Based Action Recognition with 3D Skeleton: A Survey. CAAI Trans. Intell. Technol..

[12] Ren B., Liu M., Ding R., Liu H. (2020). A Survey on 3D Skeleton-Based Action Recognition Using Learning Method. arXiv.

[13] Ramirez H., Velastin S.A., Meza I., Fabregas E., Makris D., Farias G. (2021). Fall Detection and Activity Recognition Using Human Skeleton Features. IEEE Access.

[14] Taylor C.J. (2000). Reconstruction of Articulated Objects from Point Correspondences in a Single Uncalibrated Image. Comput. Vis. Image Underst..

[15] Agarwal A., Triggs B. 3D Human Pose from Silhouettes by Relevance Vector Regression. Proceedings of the IEEE Computer Society Conference on Computer Vision and Pattern Recognition.

[16] Agarwal A., Triggs B. (2006). Recovering 3D Human Pose from Monocular Images. IEEE Trans. Pattern Anal. Mach. Intell..

[17] Xu W., Chatterjee A., Zollhöfer M., Rhodin H., Fua P., Seidel H.P., Theobalt C. (2018). Mo2Cap2: Real-Time Mobile 3D Motion Capture with a Cap-Mounted Fisheye Camera. IEEE Trans. Vis. Comput. Graph..

[18] Tome D., Peluse P., Agapito L., Badino H. XR-EgoPose: Egocentric 3D Human Pose from an HMD Camera. Proceedings of the IEEE International Conference on Computer Vision.

[19] Tome D., Alldieck T., Peluse P., Pons-Moll G., Agapito L., Badino H., De la Torre F. (2020). SelfPose: 3D Egocentric Pose Estimation from a Headset Mounted Camera. IEEE Trans. Pattern. Anal. Mach. Intell..

[20] Wang J., Tan S., Zhen X., Xu S., Zheng F., He Z., Shao L. (2021). Deep 3D Human Pose Estimation: A Review. Comput. Vis. Image Underst..

[21] Ye G., Liu Y., Hasler N., Ji X., Dai Q., Theobalt C. (2012). Performance Capture of Interacting Characters with Handheld Kinects. Proceedings of the ECCV 2012: Computer Vision—ECCV 2012.

[22] Bashirov R., Ianina A., Iskakov K., Kononenko Y., Strizhkova V., Lempitsky V., Vakhitov A. Real-Time RGBD-Based Extended Body Pose Estimation. Proceedings of the 2021 IEEE Winter Conference on Applications of Computer Vision, WACV.

[23] Zhang H.B., Zhang Y.X., Zhong B., Lei Q., Yang L., Du J.X., Chen D.S. (2019). A Comprehensive Survey of Vision-Based Human Action Recognition Methods. Sensors.

[24] Wang J., Liu L., Xu W., Sarkar K., Theobalt C. Estimating Egocentric 3D Human Pose in Global Space. Proceedings of the IEEE International Conference on Computer Vision.

[25] Chen L., Ai H., Chen R., Zhuang Z., Liu S. Cross-View Tracking for Multi-Human 3D Pose Estimation at over 100 FPS. Proceedings of the IEEE Computer Society Conference on Computer Vision and Pattern Recognition.

[26] Gholami M., Rezaei A., Rhodin H., Ward R., Wang Z.J. (2021). TriPose: A Weakly-Supervised 3D Human Pose Estimation via Triangulation from Video. arXiv.

[27] Ragaglia M., Zanchettin A.M., Rocco P. (2018). Trajectory Generation Algorithm for Safe Human-Robot Collaboration Based on Multiple Depth Sensor Measurements. Mechatronics.

[28] Saito A., Kizawa S., Kobayashi Y., Miyawaki K. (2020). Pose Estimation by Extended Kalman Filter Using Noise Covariance Matrices Based on Sensor Output. ROBOMECH J..

[29] Negin F., Koperski M., Crispim C.F., Bremond F., Coşar S., Avgerinakis K. A hybrid framework for online recognition of activities of daily living in real-world settings. Proceedings of the 13th IEEE International Conference on Advanced Video and Signal Based Surveillance (AVSS).

[30] Zhang Z., Liu Y., Li A., Wang M. A novel method for user-defined human posture recognition using Kinect. Proceedings of the IEEE 7th International Congress on Image and Signal Processing.

[31] Jalal A., Kim Y.H., Kim Y.J., Kamal S., Kim D. (2017). Robust human activity recognition from depth video using spatiotemporal multi-fused features. Pattern Recognit..

[32] Chang J., Wang L., Meng G., Xiang S., Pan C. (2018). Vision-based occlusion handling and vehicle classification for traffic surveillance systems. IEEE Intell. Transp. Syst. Mag..

[33] Dang L.M., Min K., Wang H., Piran M.J., Lee C.H., Moon H. (2020). Sensor-based and vision-based human activity recognition: A comprehensive survey. Pattern Recognit..

[34] Li X., Fan Z., Liu Y., Li Y., Dai Q. (2019). 3D Pose Detection of Closely Interactive Humans Using Multi-View Cameras. Sensors.

[35] Caron F., Davy M., Duflos E., Vanheeghe P. (2007). Particle Filtering for Multisensor Data Fusion with Switching Observation Models: Application to Land Vehicle Positioning. IEEE Trans. Signal Process..

[36] Andrieu C., Davy M., Doucet A. (2003). Efficient Particle Filtering for Jump Markov Systems. Application to Time-Varying Autoregressions. IEEE Trans. Signal Process..

[37] Doucet A., Freitas N., Gordon N. (2001). Sequential Monte Carlo Methods in Practice.

[38] Inoue M., Inoue S., Nishida T. (2018). Deep Recurrent Neural Network for Mobile Human Activity Recognition with High Throughput. Artif. Life Robot.

[39] Zhu W., Lan C., Xing J., Zeng W., Li Y., Shen L., Xie X. Co-Occurrence Feature Learning for Skeleton Based Action Recognition Using Regularized Deep LSTM Networks. Proceedings of the 30th AAAI Conference on Artificial Intelligence, AAAI.

[40] Núñez J.C., Cabido R., Pantrigo J.J., Montemayor A.S., Vélez J.F. (2018). Convolutional Neural Networks and Long Short-Term Memory for Skeleton-Based Human Activity and Hand Gesture Recognition. Pattern Recognit..

[41] Kim K., Cho Y.K. (2020). Automatic Recognition of Workers’ Motions in Highway Construction by Using Motion Sensors and Long Short-Term Memory Networks. J. Constr. Eng. Manag..

[42] Shang X., Song M., Wang Y., Yu C., Yu H., Li F., Chang C.I. (2021). Target-Constrained Interference-Minimized Band Selection for Hyperspectral Target Detection. IEEE Trans. Geosci. Remote Sens..

[43] Wang P., Wang L., Leung H., Zhang G. (2021). Super-Resolution Mapping Based on Spatial-Spectral Correlation for Spectral Imagery. IEEE Trans. Geosci. Remote Sens..

[44] Lugaresi C., Tang J., Nash H., Mcclanahan C., Uboweja E., Hays M., Zhang F., Chang C.-L., Yong M.G., Lee J. (2019). MediaPipe: A Framework for Building Perception Pipelines. arXiv.

[45] Rossi L., Paolanti M., Pierdicca R., Frontoni E. (2021). Human trajectory prediction and generation using LSTM models and GANs. Pattern Recognit..

[46] Kim K., Cho Y.K. (2020). Effective inertial sensor quantity and locations on a body for deep learning-based worker’s motion recognition. Autom. Constr..

[47] Roberts D., Calderon W.T., Tang S., Golparvar-Fard M. (2020). Vision-Based Construction Worker Activity Analysis Informed by Body Posture. J. Comput. Civ. Eng..

[48] Akhavian R., Behzadan A.H. (2016). Smartphone-Based Construction Workers’ Activity Recognition and Classification. Autom. Constr..

[49] Sam M., Franz B., Sey-Taylor E., McCarty C. Evaluating the perception of human-robot collaboration among construction project managers. Proceedings of the Construction Research Congress 2022.

[50] Chea C.P., Bai Y., Pan X., Arashpour M., Xie Y. (2020). An integrated review of automation and robotic technologies for structural prefabrication and construction. Transp. Saf. Environ..

[51] Pransky J. (2020). The Pransky interview: Dr. Tessa Lau, Founder and CEO of Dusty Robotics. Ind. Robot Int. J. Robot. Res. Appl..

[52] Cardno C.A. (2018). Robotic rebar-tying system uses artificial intelligence. Civ. Eng. Mag. Arch..

[53] Eternal Robotics MYRO. www.eternalrobotics.com/solutions/#SMyro.

[54] Putra P.U., Shima K., Shimatani K. Markerless Human Activity Recognition Method Based on Deep Neural Network Model Using Multiple Cameras. Proceedings of the 5th International Conference on Control, Decision and Information Technologies, CoDIT.

[55] Siddiqi M.H., Almashfi N., Ali A., Alruwaili M., Alhwaiti Y., Alanazi S., Kamruzzaman M.M. (2021). A Unified Approach for Patient Activity Recognition in Healthcare Using Depth Camera. IEEE Access.

[56] Agarwal P., Alam M. (2020). A Lightweight Deep Learning Model for Human Activity Recognition on Edge Devices. Procedia Comput. Sci..

[57] Antwi-Afari M.F., Qarout Y., Herzallah R., Anwer S., Umer W., Zhang Y., Manu P. (2022). Deep learning-based networks for automated recognition and classification of awkward working postures in construction using wearable insole sensor data. Autom. Constr..

